# Unraveling the complexity of basal-like breast cancer

**DOI:** 10.18632/oncotarget.314

**Published:** 2011-08-28

**Authors:** Lauren L. C. Marotta, Kornelia Polyak

**Affiliations:** Department of Medical Oncology, Dana-Farber Cancer Institute, Boston, MA; Program in Biological and Biomedical Sciences, Harvard Medical School, Boston, MA; Department of Medical Oncology, Dana-Farber Cancer Institute, Boston, MA; Program in Biological and Biomedical Sciences and Department of Medicine, Harvard Medical School, Boston, MA; Department of Medicine, Brigham and Women's Hospital, Boston, MA

Basal-like breast cancer (BLBC) is a form of breast cancer with generally poor prognosis that expresses basal cell markers and has an undifferentiated phenotype. The majority of BLBC is also classified as “triple-negative breast cancer” due to the lack of expression of the estrogen and progesterone receptors and the lack of overexpression of HER2. BLBC is the only major breast cancer subtype with no targeted therapies, and although ~20% of patients diagnosed with BLBC respond well to chemotherapy and are essentially cured, the remaining ~80% succumb to their disease in just a few years after diagnosis. Therefore, new molecular-based approaches to treating BLBC are urgently needed. Several recent studies, including our report in the July 2011 issue of *The Journal of Clinical Investigation* [1], have used large-scale, unbiased approaches to offer valuable insights into the details of complex aspects of BLBC.

In our report, we explored how intratumor heterogeneity impacts BLBC. After screening 1,576 genes, we found that the JAK2/STAT3 pathway is preferentially required in CD44+CD24− breast cancer cells, which are most common in BLBC [2]. We identified a JAK2/STAT3 inhibitor, NVP-BSK805, that decreased basal-like breast tumor growth by eliminating this cell type, showing that targeting the JAK2/STAT3 pathway is a potential way to treat BLBC. Since we also found that basal-like breast tumors include CD24+ cells, which may also play a role in breast cancer progression [3], this would likely be most effective in combination with other cell type-specific drugs.

Other researchers have also looked into the importance of intratumor heterogeneity in BLBC by taking into account the added complexity of cell plasticity. Using a high-throughput screen for inhibitors of cells that had undergone an epithelial-mesenchymal transition (EMT), Gupta *et al.* [4] discovered that the compound salinomycin inhibited basal-like breast tumor growth. Thus, therapies that target particular cellular phenotypes could be useful in treating BLBC. Corroborating this work, LOXL2 was recently found to be involved in EMT and BLBC metastasis [5].

Important BLBC discoveries have also come from looking at tumors as a whole rather than at specific cancer cell populations. Sun *et al.* [6] screened all known human kinases and phosphatases for anchorage-independent growth suppression and thereby elucidated a complicated intracellular network in triple-negative breast cancer. They found that PTPN12 normally suppresses HER2 and PDGFR-β and that inhibition of these together with lapatinib and sunitinib, respectively, greatly increased tumor growth inhibition. These results could be directly applicable to BLBC and suggest that drug combinations may often be necessary to kill the mixtures of cancer cells present in tumors.

In another study at the whole-tumor level, published in the same issue of *The Journal of Clinical Investigation* as our report, Lehmann *et al.* [7] classified 587 triple-negative breast cancer cases into six subtypes using k-means clustering of gene expression data. Two of the identified subtypes were labeled as “basal-like” subtypes, and cell line models of them preferentially responded to cisplatin in culture and in mice. By scrutinizing intertumor heterogeneity, this work identified a potential treatment for BLBC, and it highlights the significance of personalizing breast cancer therapy.

In summary, recent advances in BLBC are helping to unravel its complexity by improving our understanding of intratumor and intertumor heterogeneity, cell plasticity, and intracellular networks. Successful targeted treatment strategies for BLBC will likely be designed based on the potential targets and tumor biology details presented here as well as additional findings from the studies discussed and research inspired by them. These will likely include tumor-specific combinations of one or more drugs for multiple cell populations or states. Notably, such therapies may appear soon, as several of the inhibitors identified here, or related compounds, are already in clinical use or development.
